# An Observational Study of Honey Bee Colony Winter Losses and Their Association with *Varroa destructor*, Neonicotinoids and Other Risk Factors

**DOI:** 10.1371/journal.pone.0131611

**Published:** 2015-07-08

**Authors:** Romée van der Zee, Alison Gray, Lennard Pisa, Theo de Rijk

**Affiliations:** 1 Netherlands Centre for Bee Research, Tersoal, Netherlands; 2 Department of Mathematics and Statistics, University of Strathclyde, Glasgow, United Kingdom; 3 RIKILT Wageningen UR, Institute of Food Safety, Wageningen, Netherlands; San Diego, UNITED STATES

## Abstract

This article presents results of an analysis of honey bee losses over the winter of 2011-2012 in the Netherlands, from a sample of 86 colonies, located at 43 apiaries. The apiaries were selected using spatially stratified random sampling. Colony winter loss data were collected and related to various measures of colony strength recorded in summer, as well as data from laboratory analysis of sample material taken from two selected colonies in each of the 43 apiaries. The logistic regression model which best explained the risk of winter loss included, in order of statistical importance, the variables (1) *Varroa destructor* mite infestation rate in October 2011, (2) presence of the cyano-substituted neonicotinoids acetamiprid or thiacloprid in the first 2 weeks of August 2011 in at least one of the honey bee matrices honey, bees or bee bread (pollen), (3) presence of *Brassica napus* (oilseed rape) or *Sinapis arvensis* (wild mustard) pollen in bee bread in early August 2011, and (4) a measure of the unexplained winter losses for the postal code area where the colonies were located, obtained from a different dataset. We consider in the discussion that reduced opportunities for foraging in July and August because of bad weather may have added substantially to the adverse effects of acetamiprid and thiacloprid. A novel feature of this work is its use of postal code random effects from two other independent datasets collected in the annual national monitoring by questionnaires of winter losses of honey bees in the Netherlands. These were used to plan the sample selection and also in the model fitting of the data in this study. It should however be noted that the results of the present pilot study are based on limited data, which may consequently reveal strong factors but fail to demonstrate possible interaction effects.

## Introduction

Honey bee colony losses in the Netherlands have been above 20% during the period 2006–2013 [1–5]. This is a higher level of loss than the loss rates reported from most European countries [6]. The Dutch honey bee loss results come from analysis of the annual Winter Survival Monitoring Surveys of beekeepers carried out in the Netherlands by the Netherlands Centre of Bee Research (NCB), which use an international standardised questionnaire, i.e. the COLOSS questionnaire [2–5]. Analysis of these annual Dutch beekeeper surveys of colony losses has shown that beekeepers who treated against the ectoparasite *Varroa destructor* (referred to in this study as “varroa mite”) in winter and in summer always experienced significantly lower risk of winter losses compared with beekeepers who only treated in summer. The varroa treatment strategy is therefore an important risk factor for winter losses and an important control measure to reduce winter losses. Other factors which were regularly found to be significant were queen succession problems and the possibility for colonies to use specific sources (willow, oilseed rape, maize, heather) for foraging [2–5]. The data modelling used in the annual Dutch Winter Survival Monitoring reports [2–5] revealed considerable spatial variation in risk of winter loss between administrative (postal code) areas, after allowing for the effects of the significant predictors of loss which were recorded at beekeeper level. The models used were mostly generalized linear mixed models (GLMMs; [7]) incorporating both beekeeper and postal code area effects (random effects) as well as the fixed (specific) model factors such as varroa treatment. Spatial information thought to be potentially important, concerning land use and local use of agricultural pesticides, was not available in these studies and the effects of any such variables are included in the postal code random effects. We can therefore interpret the postal code level random effect as a measure of unexplained spatial variation in the risk of winter loss. Similarly the beekeeper level random effect is a measure of unexplained variation in the risk of winter loss corresponding to beekeeper level factors. We make use of this postal code effect in the present study. Observational studies at colony level are needed to explore how spatial level factors correspond to honey bee winter losses and can therefore explain the large variation in risk of colony winter loss between areas as reported in the annual Monitoring Survey reports. The present work is such an observational study.

Because of limited financial resources we had to develop a study design which aimed to explore the contribution of at least some of the main drivers behind colony winter loss, to facilitate the design of a more extensive and expensive longitudinal follow-up study. We considered the following possible risk factors and the timeframe in which effects may be observed which relate to winter loss:

(1) Honey bee colonies must in summer produce a population with enough lifespan to survive a sometimes long Dutch winter with substantial variation in temperatures. A severe infestation of a colony with parasites in summer, or poor weather limiting the foraging opportunities, may prevent the colony from building up a population with enough lifespan to survive winter and hence increase the risk of winter loss [8, 9].

(2) Another risk factor for colony health, explored in a growing number of scientific publications, is the role of agricultural pesticides in general and neonicotinoids in particular (see [10] for an overview). Discussions in local and national media received a wide audience, led to discussions in the Dutch Parliament [11] and were the direct motivation for the present observational pilot study. Pesticide use is high in the Netherlands, because of its important agricultural sector. In 2008, for example, 5,605 tonnes of active pesticide ingredients were used in the Netherlands [12]. The few observational surveillance studies [13,14,15] that were available at the start of this study report that honey bee matrices (honey, pollen, wax and bees) can be severely contaminated with pesticides, including neonicotinoids such as thiacloprid, imidacloprid and (the less often used) acetamiprid. These monitoring studies [14–15] mainly focus on pesticide exposure of specific crops such as oilseed rape, sunflowers and maize. Identification of a particular source of contamination may be important if a pesticide is used on a specific crop and honey bees are only exposed to it via foraging on that crop, as this might enable taking specific measures to limit risk of exposure. The main pesticides investigated in this study are however commonly used both in agriculture and by hobbyist gardeners. For example, the least toxic neonicotinoid, thiacloprid, which is considered safe for honey bees, is widely used for protection of crops and plants, including late blossoming plants such as strawberry, raspberry and species of blueberry, and not only on a few specific crops. Application of thiacloprid-based commercial products is also advised during blossoming of plants in open culture [16]. Neonicotinoids have also been found in surface water [17] and may therefore contaminate other sources foraged by honey bees, making them a potentially more important risk and one which is not easily avoided.

With these considerations in mind we chose exposure to parasites, pathogens or pesticides in the critical summer period as the basis of our study design. We investigate whether the presence of agricultural pesticides in three honey bee matrices (i.e. honey, bee bread/pollen and bees), degree of varroa mite infestation, presence of *Nosema* spp. and presence of a range of honey bee viruses in summer, as well as operation size, queen age and several measures of colony strength, are related to colony winter loss. We also examine whether or not the degree of varroa mite infestation in October is related to colony winter loss, as was found in a German study [15].

We collected data on *Nosema* spp. in July 2011, because we found in a longitudinal study in the Netherlands that *Nosema ceranae* infection was most often present in that month (unpublished NCB data). The other samples were collected during visits in the first two weeks of August, to determine effects of the above mentioned factors on the building up of the colony winter population. The timing of visits was also planned in order to co-operate with the interest of the participating beekeepers in wishing to start the varroa mite summer treatment.

Additionally we investigate whether the postal code effect, as calculated from the (large) Dutch National Survey 2012 [4] of honey bee survival over winter 2011–2012, can help to explain the variation in risk of winter loss between postal code areas in this study. The postal code effects calculated from the previous Dutch National Survey 2011 [3] were used to design a spatially stratified approach for data collection in the present study. Making use of these postal code effects in this way has the advantage of ensuring a balanced representation of high and low risk areas in the sample of apiaries selected for the present study. The method is explained below. To our knowledge this is the first colony level observational study with a study design based on spatial random effects calculated from national survey questionnaire data from beekeepers. The inclusion of random effects terms in the model fitting of our data is also done in a novel way and provides a better fitting model.

## Materials and Methods

### Ethics statement

Varroa mites, bees, beebread and honey were collected from honey bee colonies with permission of the beekeepers.

### Use of data from the Dutch surveys of honey bee survival in winter 2010–2011 and 2011–2012 in the present study

Results from the analysis of the previously collected colony loss data from the 1541 and 1673 beekeepers respectively participating in the large scale Dutch National Monitoring Surveys 2011 [3] and 2012 [4] were used to plan the sampling design for this new study and in the model fitting of the colony level data collected in the new study.

The data in these Monitoring Surveys for honey bee winter survival were obtained from the standardised COLOSS questionnaires 2011 and 2012, concerning colony winter loss in winter 2010–2011 and 2011–2012 respectively as well as colony management. The analysis used a generalized linear mixed model (GLMM; [7]) with a binomial distribution for the number of colonies lost from the wintered colonies; see also [18]. These GLMMs allow for correlated observations, i.e. colonies belonging to beekeepers within postal code areas indicated here as “PC areas”. The Netherlands can be grouped into 90 of these administrative PC areas. Colonies managed by any one beekeeper are unlikely to be lost, or not lost, independently of each other as they are subject to similar management practices. Similarly there are likely to be common factors affecting the colonies of beekeepers within any one PC area, which may well differ between areas. These beekeeper and area effects are allowed for in the model fitting, giving more statistically valid results than if the observations were treated as independent. Beekeeper and PC area were both included as random intercepts in the models [7], to allow for differences between the effects of beekeeper management and PC area. Management characteristics were specified as fixed effects (specific effects of interest) in the model. The following factors were significant in the 2012 National Survey data modelling: foraging on heather (lower risk of loss, p<0.0001), varroa treatment (treatment in summer and winter, or only winter (lower risk of loss compared with only summer treatment, p = 0.0034), reported foraging on maize (yes/no, higher risk of loss if yes, p = 0.0046), comb renewal (more renewal showed lower risk of loss, p = 0.0228) and foraging on willow in spring (yes/no, lower risk of loss if yes, p = 0.0484).

A full analysis from the 2011 Survey was not yet available at the time that the sampling for the present study was prepared and performed. The random effects (“PC 2011 effects”) were therefore calculated from a model which only included an intercept, a beekeeper random effect and a postal code random effect, rather than a final model with explanatory variables included in it as well. The model fitting used the data from 1412 responding beekeepers, all members of one of the 3 national beekeeper associations, with 12,924 colonies in total before winter 2010–2011, who responded before June 15 2011 and who provided valid information about winter losses and postal code information.

**Fig 1:**
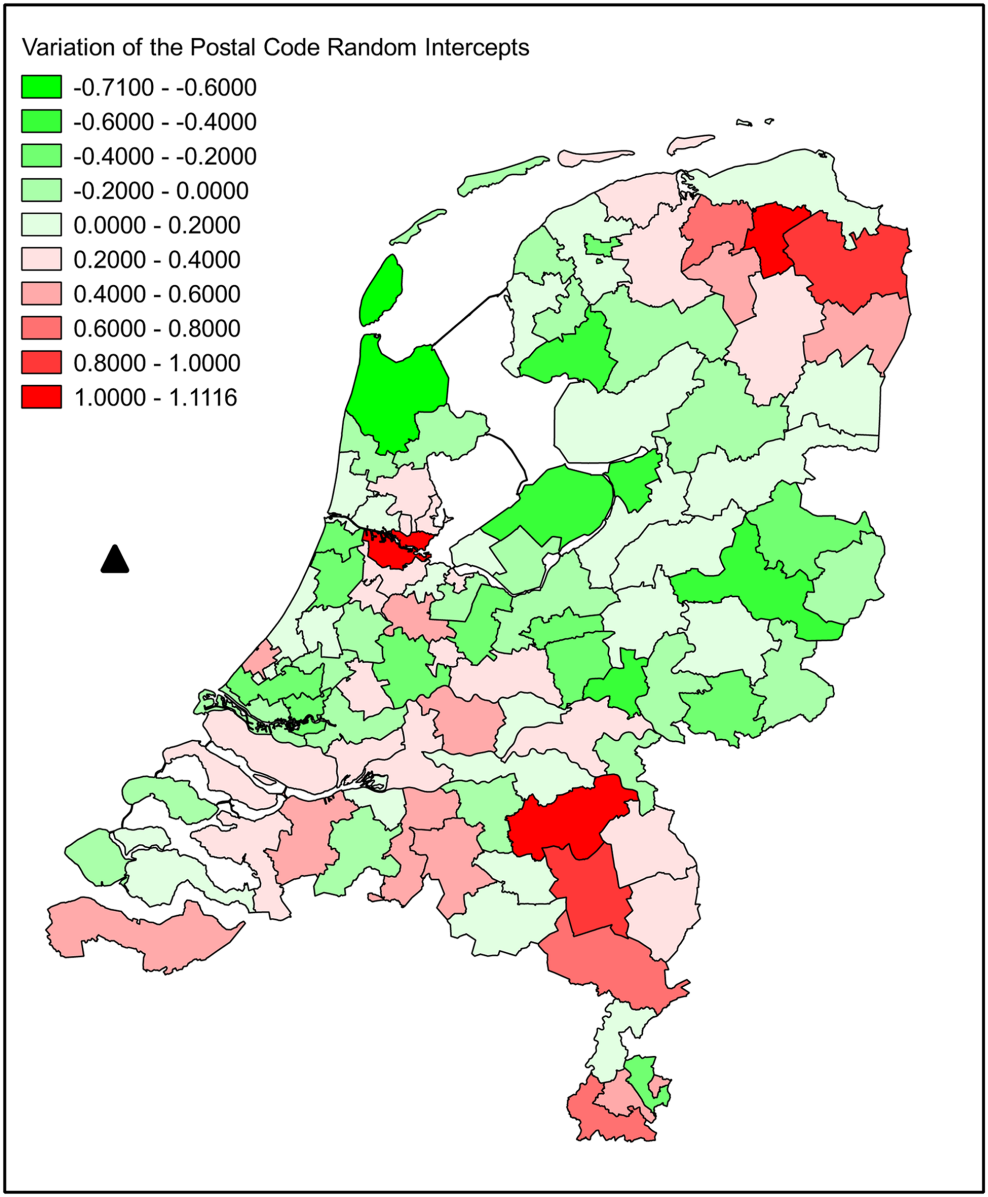
Choropleth map showing the spatial variation in the postal code level random effects. These effects were estimated from a binomial GLMM allowing for significant fixed effects for beekeeper risk of colony loss in the Netherlands and also with a beekeeper random effect, for the 1 to 50 colony operations used in the model fitting. The data are derived from the Dutch National Monitoring Survey for Honey Bee Winter Loss 2012. The legend within the figure shows the key to the colour coding. Darker green indicates areas of lower risk and darker red areas of higher risk of loss. The value of the postal code effect was used as a predictor in the final model of the data in the present study.

We used the values of these postal code level random intercepts (PC 2011 effects), referred to here generally as “postal code effects”, as derived from the Monitoring Survey 2011, to select the beekeeping operations for the present study. We also used the corresponding postal code level random intercepts (“PC 2012 effects”) from the analysis of the Monitoring Survey 2012 as a fixed effect (a covariate) in generalized linear regression models (GzLMs [7]). See the Sampling scheme and Statistical analysis paragraphs below for further details. In a different context, the authors in [19] discuss the benefits of using random intercepts derived from one model as values of a predictor variable in another model. Fig 1 shows these PC 2012 postal code effects from the model fitting using the data from the Dutch Monitoring Survey 2012. It can be seen that there is considerable spatial variation between the PC areas in these effects representing unexplained risk of winter loss. (A similar map is available in [4], but, as this is a report in Dutch not readily accessible to an international readership, Fig 1 is provided here for clarity).

### Sampling scheme

The values of the PC area random effects calculated in the mixed model fitting of the Dutch Monitoring Survey 2011 data for losses over winter 2010–2011 [3] were used to split the beekeeper operations into two sub-groups. Only beekeepers thought from the Monitoring Survey data to have a minimum of 5 surviving colonies after winter were considered to be certain to have at least 2 colonies available for sampling. These beekeepers were divided into those in PC areas with a negative postal code effect (indicating a lower risk of winter loss in that area in winter 2010–2011) and those in PC areas with a positive postal code effect (indicating a higher risk of winter loss). A randomised sample of 43 operations was taken, with 21 operations randomly selected from the areas identified in this way as having a lower (negative) postal code effect and 22 operations randomly selected from areas with a higher (positive) postal code effect. This strategy was used to ensure a balanced representation of beekeeper operations from areas determined to be higher and lower risk areas for winter losses. The level of risk could not be fully explained from effects included in previous model fitting. As these areas with different levels of risk are assumed to vary in some other, as yet unexplained, way, it was considered to be important that both high and low risk areas were well represented in the sample. The strategy used was a simple way to ensure that this was achieved. In fact one selected beekeeper had 3 colonies rather than 5 or more. From each beekeeping operation, 2 colonies were randomly selected and included in the study sample (sample size n = 86). Participating beekeepers were instructed that they should not migrate these colonies during the study period, which began on 1^st^ July 2011.

Sampling of the selected colonies and processing of the samples was carried out as follows:

1. **During the last week of July 2011** a minimum of 35 forager bees were collected from the closed hive entrance between the hours of 11.00 and 14.00. Samples were conserved in situ in ethanol 70% and sent for analysis at the Warsaw University of Life Sciences, faculty of Veterinary Medicine, Department of Pathology and Veterinary Diagnostics Laboratory of Bee Diseases, Poland, for analysis of *Nosema* spp. presence, and band strength of both *Nosema ceranae* and *Nosema apis*.
2. **During the first 2 weeks of August 2011** a sample of 50 bees was taken– 25 from frames with brood and 25 from frames without brood—for pesticide residue analysis. The samples were stored in situ on dry ice (-80°C) and conserved at this temperature for a maximum of 2 days before arriving at the RIKILT laboratory at Wageningen UR, where all samples were conserved at –20°C until analysis.
3. A minimum of 30 bees were sampled in an identical way for virus analysis. These samples were stored in situ on dry ice (-80°C) and sent on dry ice to the Food and Environment Research Agency in York, England, for analysis.
4. Further a minimum of 50 cells with bee bread were sampled. Half of the sample was used for pesticide analysis by RIKILT, the other half for palynological determination by the Animal Ecology Team of ALTERRA Wageningen.
5. Mature and half-ripe honey was sampled from each colony for pesticide residue analysis, stored in situ on dry ice (-80°C) and stored at –20°C until analysis by RIKILT.
6. **During the last week of July 2011 and last week of October 2011** approximately 200–250 bees per colony were sampled by the beekeeper and sent to the Nederlands Centrum Bijenonderzoek (NCB) for determination of the level of varroa mite infestation.

### Statistical analysis

Statistical analysis was performed using the software packages R [20] and SPSS 20. Descriptive statistics, statistical tests and generalized linear modelling were used. The bar charts were produced with Excel. The map was produced with QGIS version 2.0.1-Dufour (available at http://www.qgis.org/en/site/index.html). Correlation between operation size (the total number of wintered colonies of the participating beekeeper at October 1 2011) and number of *V*. *destructor* mites per 100 bees (varroa mite infestation rate) was calculated using the Pearson correlation (appropriate for data that is approximately (bivariate) normally distributed). Spearman’s rank correlation (suitable for non-normal continuous data) was used to calculate the correlation between postal code area random effect and varroa mite infestation rate. Here p-values less than 0.05 are considered significant. A two-sample Mann-Whitney U-test was used to test for differences in the level of postal code area random effect between two groups defined by a binary predictor variable (presence of a pesticide or plant species in bee bread).

We used a logistic GzLM to determine associations between risk of colony overwinter mortality in the sampled population and the predictor variables operation size, colony size, number of frames with brood, varroa mite infestation rate, presence of various pesticides in honey bee matrices, presence of plant families in pollen and the postcode area random effects from the National Monitoring Survey 2012. In this GzLM model, the number of colonies dead in spring 2012 was taken as the dependent variable and was assumed to follow a binomial distribution. As the number of colonies studied for each selected beekeeper was the same and small (2 colonies), it was not thought necessary to allow for possible extra-binomial variation in the data. First, single factor models were fitted using the different variables of interest as a single predicting factor or covariate to identify those variables which were associated with winter losses. The postal code (PC) area effects from the National Monitoring Survey 2012 were used as one of these predictor variables for winter loss. This was done because these postal code effects were taken as a measure of the risk of loss that could not previously be explained in the much larger dataset of the National Monitoring Survey 2012. The variables which were accounted for in that model were varroa mite treatment, percentage renewal of brood comb, reported foraging on maize (widespread in the Netherlands) and reported foraging on heather (confined to specific areas of the Netherlands). Both the present dataset and the National Monitoring Survey 2012 relate to losses over winter 2011–2012. Next, the best fitting multivariable model was determined by including all the variables which were significant in the single variable models and which remained significant in the multivariable model. We examined the AIC (Akaike’s Information Criterion) for the model when each term was dropped, one at a time, and the corresponding likelihood ratio test (LRT) statistic and its p-value. A rise in the AIC when the term was dropped, together with a significant LRT p-value, indicates that that term should not be dropped from the model as it does significantly contribute to explaining the risk of colony loss.

### Chemical analysis

#### Reference standards

Most of the reference standard ingredients were purchased from commercial suppliers. The imidacloprid metabolites were donated by Bayer Crop Science (S1 Table). A combined solution was prepared by mixing different volumes of stock solutions of the individual pesticides until the target concentration of the mix stock solution was obtained (S1 Table). Acetonitrile, methanol, (all of HPLC grade or better), and HPLC grade water were purchased from Biosolve (Valkenswaard, The Netherlands). Acetic acid, sodium sulphate, and magnesium sulphate were obtained from Merck (Darmstadt, Germany), and formic acid and ammonium formate were obtained from Sigma-Aldrich (Zwijndrecht, The Netherlands).

#### Extraction

*Bees*: 1.0 (± 0.05) g of frozen sample material was weighed into a 50 mL Greiner tube. Extraction was performed by adding 3.0 mL of water, directly followed by 4.0 mL of extraction liquid (acetic acid/acetonitrile = 1/99 (v/v)). The sample was homogenised for 1 minute with the aid of an ultra-turrax, followed by addition of 1.6 g of MgSO_4_ and 0.40 g of sodium acetate. After homogenisation (vortex for 30 s) and centrifugation (5 minutes at 2000 rcf), 250 μL of extract was diluted with 250 mL of water and filtered with the aid of a 0.45 μm syringeless filter device (Mini-UniPrep, Whatman, Forham Park, NJ), resulting in a matrix equivalent in the extract of 0.125 g/mL.

*Honey/Bee bread*: The extraction procedure for honey or bee bread was identical to the procedure as described for bees. However, it was not necessary to use the labour intensive step of ultra-turrax homogenisation as honey and bee bread sample material disintegrates when mixed with water before the extraction liquid is added. This step was replaced by shaking end-over-end for 30 minutes.

#### Liquid Chromatography Tandem Mass Spectrometry (LC-MS/MS) analysis

For LC-MS/MS analysis a Shimadzu high performance liquid chromatography (HPLC) system (Shimadzu- ‘s Hertogenbosch, the Netherlands) and an Applied Biosystems 5500 triple quadrupole mass spectrometer (Applied Biosystems Bleiswijk, the Netherlands) equipped with an electrospray (ESI) source were used for determination of pesticides. Separation was performed on a 100 mm × 3.0 mm i.d., 3.0 μm Atlantis T3 C-18 column (Waters, Etten-Leur, the Netherlands) using a flow rate of 0.40 mL/minute. The column temperature was maintained at 35°C. Eluent A was water containing 5 mM ammonium formate and 0.1% (v/v) formic acid. Eluent B was water/methanol 5/95 (v/v) containing 5 mM ammonium formate and 0.1% (v/v) formic acid. The HPLC gradient started with 100% A for 0.5 minutes, was linearly increased to 100% B over 4.0 minutes, and kept at this percentage for 4.5 minutes. Finally, the gradient was switched to 100% A again over 0.5 minutes and equilibrated for 4.0 minutes before the next injection took place. The injection volume was 10 μL.

#### MS/MS conditions

ESI-MS/MS was performed using scheduled multiple reaction monitoring (sMRM) in positive mode and multiple reaction monitoring (MRM) in negative mode. In sMRM mode the detection window was set to 90 s and the target scan time was set to 0.5 s. In MRM mode acquisition was done with 10 ms dwell time. For both scan types the settling time and pause time was set to 5 ms. The number of data points across the peaks was at least eight. The settings of the ESI-source were as follows: source temperature 300°C, curtain gas 20 psi, source gas 1 60 psi, source gas 2 60 psi, ion spray voltage -2000 V neg. mode and + 5500 V pos. mode and collision gas (nitrogen) medium. The analyte-dependent parameters declustering potential (DP), collision energy (CE) and cell exit potential (CXP) are listed in S2 Table.

#### Verification of recovery and matrix effects

For verification of recovery for the different extraction methods, each matrix was fortified at levels given in S3 Table. In addition, one non-fortified sample was included in the test set. The extract of the non-fortified sample was also used for preparation of a matrix-matched calibration curve. In the LC-MS/MS sequence, for each matrix, the five sample extracts were bracketed by the matrix-matched calibration standard (and a solvent standard at the same concentration). Average recoveries and relative standard deviations (RSDs) were calculated for the fortified samples against matrix-matched standards. Recoveries obtained therefore reflect the recovery from the extraction procedure (S3–S5 Tables).

#### Validation

To all three matrices known quantities of the pesticides which were to be investigated were added in 5-fold at three levels (1x, 2x and 10x spiking level (SL), S3–S5 Tables). Limits Of Quantification (LOQ) were set at the lowest spiking level, with the exception of imidacloprid urea and thiacloprid in pollen for which higher LOQs of 1.0 and 2.0 μg/kg respectively were established. For certain pesticides it was feasible to determine a detection limit (Limit Of Determination, LOD) which was lower than the LOQ and complied to conditions for the defined analytical parameters ion ratio and retention time [21]. Pesticides present at the level of LOD<x<LOQ were reported as “trace”. Matrix-matched calibration curves were constructed by diluting the combined stock solution (S1 Table) 500, 2000, 5000, 20000, and 40000 times with extract of blank honey, bees, and stored pollen. These standards were analysed for verification of linearity of response versus concentration. In the LC-MS/MS sequence, the 2000 times diluted matrix-matched standard was repeatedly analysed every 5–8 injections. Recoveries were calculated based on one-point matrix-matched calibration, using the average of the 2000 times diluted matrix-matched standard preceding and following the sample (bracketing).

### Analysis of the presence of pathogens

#### Varroa mite infestation rate

The proportion of phoretic mites per 100 bees (mite infestation rate) was estimated following OIE protocol [22].

#### Virus analysis

Real-time PCR was carried out with the suite of TaqMan assays described in Chantawannakul et al. (2006) [23] which enabled the detection of: *chronic bee paralysis virus* (CBPV), *acute bee paralysis virus* (ABPV), *Apis iridescent virus* (AIV), *Kashmir bee virus* (KBV), *sacbrood virus* (SBV), *deformed wing virus* (DWV), *black queen cell virus* (BQCV) and *Israeli acute paralysis virus* (IAPV).

#### *Nosema* spp. analysis

The abdomens of the collected honey bees were crushed in Eppendorf tubes using liquid nitrogen and sealed pipette tips. 0.5 ml of distilled water was added to each tube and mixed with the powdered abdomens. A pooled sample was made by taking 0.1 ml of the solution from each tube. This pooled sample was centrifuged for 6 minutes at 800g. The pellet was then subjected to DNA extraction using a Dneasy Plant Mini Kit (Quiagen) according to the manufacturer’s protocol. Extracted DNA samples were stored at -20°C until further processing. Thermal setup and all other PCR conditions were implemented according to [24]. Electrophoresis of the PCR product was performed in 1.5 agarose gel stained with ethidium bromide. Then the product was visualised under UV light. If the result was positive we examined the individual bees from the remaining samples using light microscopy. A quantity of 2 ml of the spore solution was placed on an IFI slide and the presence of spores was checked.

### Palynological determinations

Bee bread was collected from approximately 25 honey comb cells to analyse the pollen composition of each sample. The pooled content of these cells was thoroughly mixed and stored in containers with 70% ethanol. Samples for microscopic inspection were prepared from the stored mixed pollen bread by dissolving a small subsample of the mixture in 2 ml water. Two drops of this pollen solution were placed on an observation glass and mounted in glycerine jelly containing basic fuchsin to stain the pollen grains. Identification took place under a light microscope at 400 times magnification with the assistance of a reference pollen collection of approximately 130 species and reference documents (see [25]). For each sample we estimated the percentage contribution of each pollen taxon to the total number of pollen grains. Pollen taxa were identified to the lowest possible taxonomic level.

### Colony characteristics

During the August 2011 apiary visit, various colony characteristics were assessed for all 86 sampled colonies. These were (1) colony size (number of frames with bees), (2) size of the brood nest (number of frames with brood), (3) presence of pollen above the brood nest (categorised as good, reasonable, moderate, bad), (4) presence of pollen (yes/no) in the frames beside the brood nest and (5) age of the queen. The sampled colonies were checked for visible signs of bee diseases.

The participating beekeepers were asked in January 2012 and April 2012 about the number of colonies lost between September 1 2011 and December 31 2011 and between January 1 2012 and April 1 2012 respectively. Case definitions as provided in the COLOSS BEEBOOK [26] were used to determine whether a colony was lost.

## Results

### Lost colonies

Of the 86 colonies in the study sample, 25 (29.1%) died between September 1 2011 and April 1 2012. Nine colonies (10.5%) died in the period between September 1 2011 and December 31 2011. Between January 1 2012 and April 1 2012 another 16 (18.6%) colonies were lost. From fitting GzLMs, no association was found between the risk of loss in the study sample and operation size (p = 0.681), although the result of the random sampling was that only one beekeeper in the sample had more than 50 colonies. A strong relationship was found between risk of colony loss (0, 1 or 2 colonies lost) for the 2 colonies observed for beekeepers in the study sample and risk of loss for those beekeepers during winter 2011–2012 as calculated from the data from the Dutch National Monitoring Survey 2012 (p<0.0001). This analysis was done to confirm that the sample of colonies of beekeepers selected for this study was consistent with the much larger sample used in the Monitoring Survey 2012. No associations (p = 0.806) were found between the number of frames with brood in August and colony winter loss in the sampled colonies, nor for the number of frames occupied with bees (decreasing risk for increasing number of frames with bees, p = 0.155), although this last effect might point to better survival for larger colonies.

### Colony characteristics

In total 68 of 86 colonies received supplemental sugar solutions before or during the visit in the first 2 weeks of August. The variation between beekeepers in terms of the exact time point at which a shortage of food reserves was observed, and therefore in starting supplemental feeding, hindered an unbiased comparison of food reserves between colonies. In 5 colonies both honey and pollen were absent. Pollen presence was estimated as good in 5, reasonable in 14, moderate in 27, nearly absent in 33, and absent in 7 colonies. No significant associations were found between differences in degree of pollen presence above the brood nest (p = 0.385), or to what extent pollen was present in the frames next to the brood nest (p = 0.887) as observed in the first 2 weeks of August 2011, and risk of colony loss. No effect was found relating age of the queen and risk of loss (p = 0.921), which was in accordance with the findings in the National Monitoring Survey 2012.

### Varroa mite related effects

No associations were found between risk of winter loss (p = 0.554) and the varroa mite infestation in the last week of July. The varroa mite infestation rate in the last week of October could be calculated for 81 of the 86 colonies in the study sample. For the other 5 colonies, data was missing for 3 colonies which died before the sampling in the last week of October took place, and for another 2 because one beekeeper could not be contacted in this period. A strong positive association (p<0.001) was found between increasing mite load in October and increasing risk of colony loss, which confirms the outcome of other studies [15, 27]. There was no significant correlation between varroa mite infestation rate and operation size (p = 0.597).

### Associations between pesticide presence and losses

In 15 colonies, trace amounts of neonicotinoids were present at levels exceeding the Limits Of Detection (LOD) but below the Limits Of Quantification (LOQ). These observations were considered as positive samples and were allocated a value of 0.05, considered to be sufficiently small for the purposes of the analysis.

Imidacloprid (including its metabolite imidacloprid desnitro), acetamiprid and thiacloprid were found in bee bread, honey or bees of 37 colonies in the study sample (n = 86). No other neonicotinoids screened for were detected (see S6 Table).

The chemical synergist piperonyl-butoxide was found in the bee bread of 6 colonies. Fipronil, fipronil-sulphide, fluvalinate-tau and propiconazole were only detected in low numbers of matrices. A remarkably high value (371 μg/kg) for propiconazole contamination was found in one of the samples (see S6 Table for more detailed descriptive information).

The statistical analysis was limited to the group of neonicotinoids rather than all the pesticides, because only this group was present in a sufficient number of samples (S6 Table).

First we considered the effect of the presence of any of several neonicotinoids (acetamiprid, imidacloprid (or imidacloprid desnitro) or thiacloprid) in colonies, compared to colonies where no neonicotinoids of any kind were found. The odds of colony loss in the group of colonies (n = 37) in which the neonicotinoids acetamiprid, imidacloprid (or imidacloprid desnitro) or thiacloprid were present in one or more matrices (bee bread, honey and/or bees), were significantly higher (p = 0.014) compared with colonies (n = 49) without the presence of any neonicotinoids.

We also tested the effect on the risk of loss of the presence of any of these neonicotinoids in colonies for each matrix separately. Presence of these neonicotinoids in bee bread (n = 24) was not associated (p = 0.209) with higher odds of loss compared to colonies without any neonicotinoids in bee bread (n = 55). However a strong association was found between higher odds of loss and presence of these various neonicotinoids in honey (n = 23, p = 0.002) rather than absence (n = 57). We also found higher odds of loss corresponding to their presence in bees (n = 14, p = 0.014) compared to colonies without any neonicotinoids (n = 70) in bees.

Next we considered the presence in at least one of the matrices of the substances thiacloprid, acetamiprid and imidacloprid singly, and again examined the effect on the risk of loss compared to 49 colonies in which no neonicotinoids of any kind were present.

Colonies (n = 30) in which thiacloprid was present in at least one matrix had significantly higher odds of loss (p = 0.009) than colonies with no neonicotinoids at all. The higher observed odds of loss if acetamiprid was present in at least one of the matrices (n = 11), compared to colonies with no neonicotinoids, was not significant (p = 0.065). The effect of the presence of imidacloprid (or imidacloprid desnitro) in at least one matrix (n = 10), compared to colonies with no neonicotinoids, was far from significance (p = 0.410). The risk of early losses (before December 31) was also significantly higher (p = 0.0461) if thiacloprid was present in colonies (in bees, honey or bee bread) compared with colonies with no neonicotinoids.

Presence of thiacloprid in honey (n = 21) was also associated (p = 0.007) with higher odds of loss compared with colonies without any neonicotinoids. Such effects were also found for presence of thiacloprid in bees (n = 10, p = 0.003) and bee bread (n = 19, p = 0.039), compared to colonies with no neonicotinoids.

**Fig 2:**
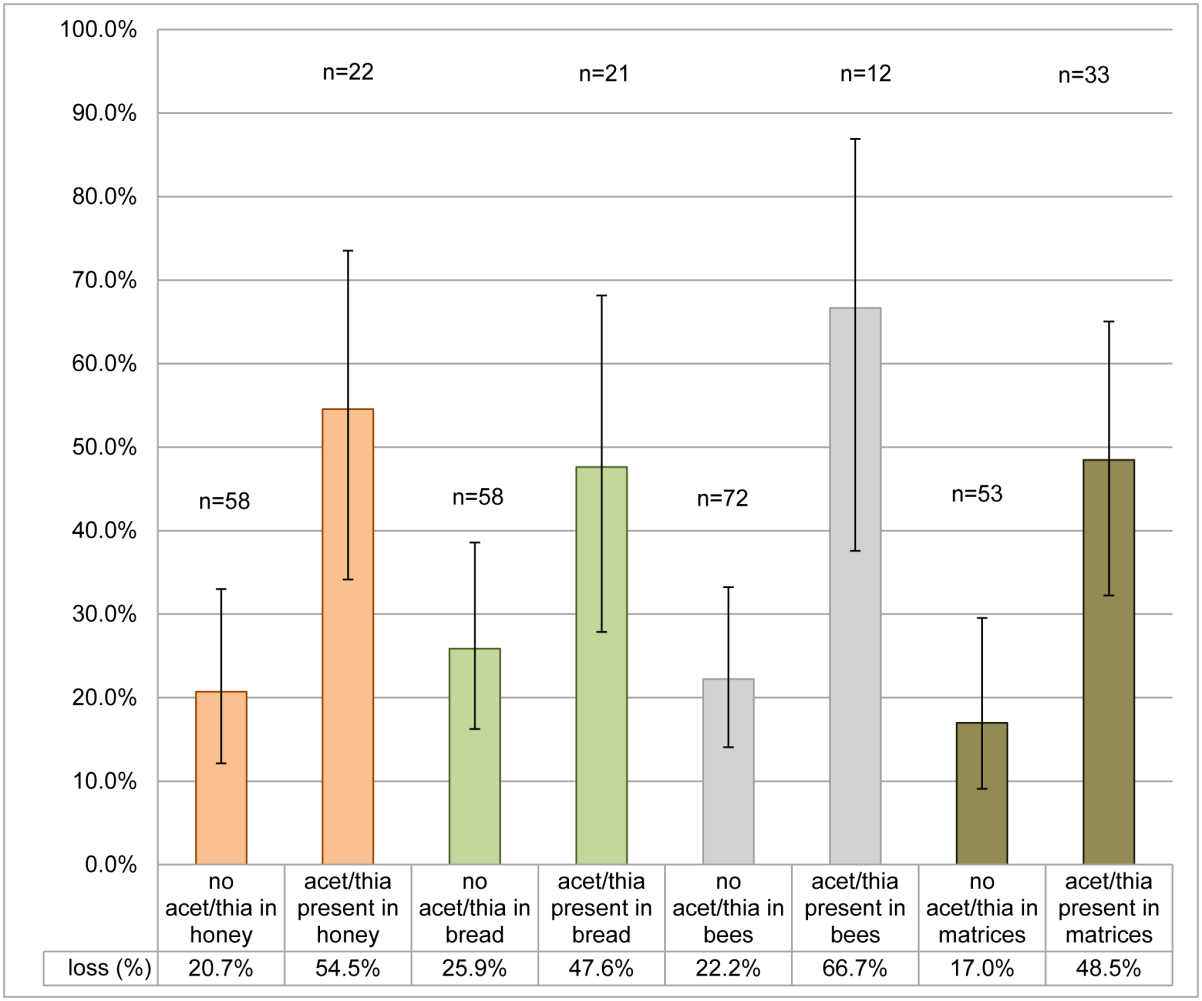
Percentage of colonies lost, with 95% confidence intervals, for colonies with/without acetamiprid or thiacloprid present.

We found that when acetamiprid was present usually (in 8 of 11 cases) thiacloprid was also present. These two cyano-substituted neonicotinoids have a similar chemical structure, different from that of the N-nitroguanidine imidacloprid [28, 29]. We therefore created a new pooled factor ‘acetamiprid or thiacloprid present (yes/no)’, indicating whether or not any of the honey, pollen and bee matrices were contaminated with thiacloprid and/or acetamiprid. This pooling should also optimise the statistical validity of the estimated model effects since relatively few colonies had acetamiprid present (and this may explain why the effect of acetamiprid considered on its own was almost but not quite significantly associated with the odds of colony loss). If thiacloprid or acetamiprid was present (n = 33), odds of colony loss were significantly higher (p = 0.0025) compared with all colonies (n = 53, including those with imidacloprid present) in which thiacloprid or acetamiprid were not detected in any of the three matrices. Fig 2 shows the percentage losses, with 95% confidence intervals, for colonies with and without either acetamiprid or thiacloprid present. There are clearly higher losses when one or both of these are present in bees, in honey, or in any of the three matrices.

### Losses related to plant species

**Fig 3:**
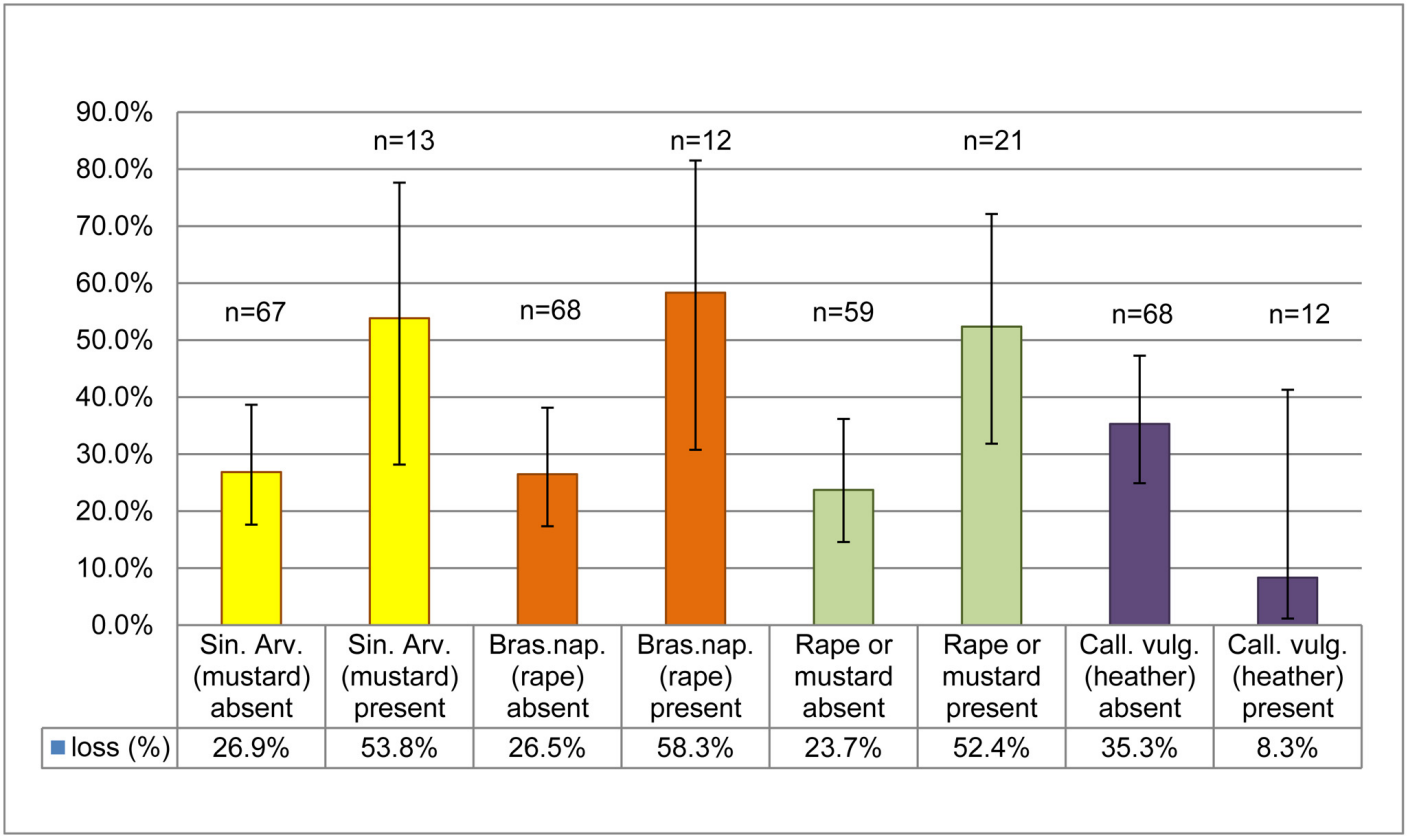
Percentage of colonies lost, with 95% confidence intervals, for colonies with/without plant species present in bee bread.

Presence of *Brassica napus* (oilseed rape) in bee bread (n = 12) was associated with higher odds of loss (p = 0.0358) compared with colonies (n = 68) where it was absent. Presence of *Sinapis arvensis* (field mustard) in bee bread (n = 13) was observed to be associated with higher odds of loss compared with colonies (n = 67) where it was absent, however this was not significant (p = 0.0627). Although we observed both *Brassica napus* and *Sinapis arvensis*, it was difficult to differentiate between these in bee bread. We therefore decided to use presence or absence of *Brassica napus* or *Sinapis* as one model factor indicated as rape/mustard. Rape/mustard in bee bread (n = 21) was also associated with higher odds of colony loss (p = 0.0179) compared with colonies in which it was not found (n = 59). No association was found between presence of maize in bee bread (n = 8) and risk of loss (p = 0.9903) compared with 72 colonies in which it was not found. Presence of *Calluna vulgaris* in bee bread (n = 12), compared with the 68 colonies in which it was not found, was not significantly associated with lower odds of loss (p = 0.0954). Fig 3 shows the percentage losses, with 95% confidence intervals, for colonies with and without these plant species present in bee bread.

### Virus and *Nosema* spp. related losses

Only the viruses BQCV, SBV, DWV, ABPV were found in the bees, which were sampled in July. It was possible to perform an analysis for all 86 colonies. We investigated associations between the virus loads and loss. No significant associations were found; BQCV (p = 0.907), ABPV (p = 0.876), DWV (p = 0.353), SBV (p = 0.719). Interactions between these viruses and other factors in the model were not found to be significantly related to the risk of loss.

We found no significant associations with a GzLM using the presence/absence of *Nosema ceranae* and/or *Nosema apis* in 86 colonies in July as a categorical model factor and relating this to the risk of colony loss (*N*. *apis*, n = 1, p = 1; *N*. *ceranae*, n = 40, p = 0.464; *N*. *apis* and *N*. *ceranae*, n = 29, p = 0.493), using no *Nosema* spp. (n = 16) as the baseline category. No significant factors were found either, when we investigated pcr band strength of the most prevalent *Nosema* species, *N*. *ceranae* in July, as a categorical factor in a GzLM and related it to risk of loss (absent (n = 17, p = 0.704), low band strength (n = 11, p = 0.704), medium band strength (n = 15, p = 0.449), high band strength (n = 43, baseline category)), in the colonies in July. Interactions between *Nosema* spp. and other factors in the model were not found to be significantly related to risk of loss.

Detailed information on the virus and *Nosema* spp. data is available in the S1 Dataset.

### Postal Code (PC) area related losses

From a GzLM, a strong relationship was found (p = 0.002) between the PC effects (from the Dutch National Monitoring Survey 2012) attributed to the colonies in those PC areas and the odds of loss. Higher odds of colony loss corresponded to higher more positive PC effect values. Of course individual beekeeper effects impact on the risk of colony loss and will vary within any one PC area with its own overall PC effect. The variation in losses between beekeepers is quantified as the beekeeper random effect in the analysis of the Dutch National Monitoring Surveys. The random sample used in the present study contained 29 apiaries with a beekeeper effect whose sign agreed with the sign (positive or negative) of the PC effect, both effects being calculated from the National Monitoring Survey 2011, but 7 apiaries with a negative beekeeper effect were located in PC areas with a positive PC effect and 7 apiaries with a positive beekeeper effect were in PC areas with a negative effect. Only 1 of the 25 lost colonies was located in an area with a negative postal code effect (suggesting lower risk of loss).

### Associations between Postal Code (PC) area effects and other variables

We also examined associations between the PC effects and other variables considered as possible predictors of colony loss. A significant though weak positive Spearman correlation of 0.30 was found between the varroa mite infestation rate in October and the attributed PC effect (p = 0.0069). From a Mann-Whitney test of the difference in PC effect, the presence of thiacloprid or acetamiprid in one of the matrices corresponded to a higher, more positive value of the PC effect (p = 0.0265). The same test indicated that heather (*Calluna vulgaris*) in bee bread was significantly associated (p = 0.0087) with higher presence in areas with lower, more negative PC effects. Maize in bee bread was also significantly associated (p = 0.0131) with higher presence in areas with lower, more negative PC effects. However rape/mustard in bee bread, which is used as a predictor of colony loss below, was not significantly associated with the PC effect.

### Most parsimonious model

We fitted several multivariable models (GzLMs), using colonies for which data was available on all of the relevant variables. Of the 86 colonies in the study, sufficient data was available for 75 colonies (see S2 Dataset). We first constructed a full model using the data from these 75 colonies and included the variables (1) varroa mite rate, (2) contamination with the cyano-substituted neonicotinoids acetamiprid or thiacloprid (yes/no) in honey, pollen or bee matrices, (3) rape/mustard present in bee bread (yes/no), and (4) the attributed PC effects (from the National Monitoring Survey 2012), listed in order of their importance in the final model. Of the 4 single variable models (Table 1), the model containing varroa mite rate improves the fit most compared to the null model and so explains more of the variation in the loss rate data than any of the other variables considered alone. Note that while the PC effect just misses being significant at the 5% level in Table 2, dropping it from the model results in a significantly less good fit (Table 3), therefore we retained it in the model.

**Table 1:** Summary results of model fitting.

Model	AIC^(1)^	DF^(2)^	^(3)^Residual Deviance
Null (intercept only) model	92.766	74	90.766
Model with only acetamiprid/thiacloprid as a factor	90.090	73	86.090
Model with only rape/mustard in bee bread as a factor	89.432	73	85.432
Model with only PC 2012 effect as a covariate	83.916	73	79.916
Model with only varroa mite rate (October) as a covariate	80.237	73	76.237
Full model M1 with all 4 significant factors	71.789	70	61.789
Full model M2 replacing acetamiprid/thiacloprid in M1 with any neonicotinoids	72.904	70	62.904
Full model M3 replacing acetamiprid/thiacloprid in M1 with thiacloprid	74.322	70	64.322
Full model M4 replacing acetamiprid/thiacloprid in M1 with acetamiprid	76.426	70	66.426
Full model M5 replacing acetamiprid/thiacloprid in M1 with imidacloprid	75.456	70	65.456

^(1)^ AIC = Akaike’s Information Criterion,

^(2)^DF = Degrees of Freedom,

^(3)^Residual Deviance = -2 logLik; a low AIC, and low Residual Deviance indicate a better model.

**Table 2:** Risk factors for colony loss: results from the best explaining model for the 75 colonies used.

Predictor variables	N. pos.^(1)^	OR (CI) ^(2)^	Slope^(3)^	SE^(4)^	Z-test^(5)^	p-value
Varroa mite rate Oct. 2011		1.17 (1.04–1.31)	0.155	0.060	2.588	0.010
Presence acetamiprid or thiacloprid	30	4.82 (1.25–18.59)	1.573	0.688	2.285	0.022
Rape/mustard in bee bread	20	5.38 (1.24–23.30)	1.683	0.748	2.251	0.024
PC 2012 effect		6.01 (0.98–36.96)	1.794	0.926	1.937	0.053

^(1)^ Number of positive cases,

^(2)^ Odds Ratio with 95% confidence interval,

^(3)^ Slope parameter,

^(4)^ Standard Error,

^(5)^ Z-test statistics

**Table 3:** Analysis of the best explaining model M1.

Term dropped	DF^(1)^	^(2)^AIC	^(3)^LRT	p-value
None		71.789		
Varroa mite rate	1	77.673	7.8835	0.004989**
Presence of acetamiprid or thiacloprid	1	75.661	5.8710	0.015392*
Rape/mustard in bee bread	1	75.300	5.5105	0.018903*
PC 2012 effect	1	74.197	4.4072	0.035787*

^(1)^ DF = Degrees of Freedom for the term dropped,

^(2)^AIC = Akaike’s Information Criterion,

^(3)^LRT = Likelihood Ratio Test statistic for the change in model fit and the p-value of the test.

Signif. codes: 0

'***' 0.001

'**' 0.01

'*' 0.05 '.' 0.1 ' ' 1

Table 1 shows the fit of the various models. The AIC decreased from 92.766 in the null model with no explanatory variables to 71.789 in model M1 including all 4 variables, and the residual deviance decreased from 90.766 to 61.789. These 4 variables were all significantly associated with risk of winter loss (Tables 2 and 3). Table 3 shows the AIC when each term is dropped one at a time from the final 4 variable model to give a 3 variable model, and the corresponding likelihood ratio test (LRT) statistic and corresponding p-value. The results indicate that all of the terms should be retained in model M1.

Additionally we fitted two other full models for comparison with model M1. In model 2 (M2) we tested the effects of neonicotinoids as a specific group by replacing the pooled acetamiprid/thiacloprid factor (2) above with the factor any (of the three) neonicotinoids (yes/no) in any of the three matrices. In model 3 (M3) we tested the effect of thiacloprid singly, by replacing factor (2) with the factor thiacloprid (yes/no) in any of the three matrices.

The best fitting model to the data is the first model M1 with an AIC of 71.789 (Table 1). In this model the term for acetamiprid/thiacloprid was highly significant (p = 0.02229). Model M2 including presence of any neonicotinoids fits almost as well, with an AIC of 72.904. The term for any neonicotinoids is also significant (p = 0.03981). Model M3 containing presence of thiacloprid is next best, but thiacloprid is not significant itself (p = 0.07667).

For comparison, we also examined the fit of a further two models, replacing factor (2) in model M1 with presence of acetamiprid (yes/no) in model M4 and with presence of imidacloprid (yes/no) in model M5. Both models explain less of the variability in the data (Table 1) than models M1, M2 and M3, and the presence of either pesticide alone is not significant at all (p = 0.1389 for imidacloprid and p = 0.2554 for acetamiprid) given the other terms in the model. This confirms the earlier results.

As for model M1, we tested the effect of dropping each term in models M2–M5, one term at a time, which enabled assessment of the PC area effect in particular. In all cases the improvement due to including the PC area effect in the model was significant. Dropping the PC effect from model M3 led to the thiacloprid factor becoming significant (p = 0.04465) owing to the smaller standard errors of the estimated effects in the simpler model. Similarly dropping PC effect from model M1 led to acetamiprid/thiacloprid becoming more significant (p = 0.01725 compared to p = 0.02229 in M1).

We therefore take model M1 as the final model. This 4 variable model was found to be the best fitting and most parsimonious model available to describe the risk of colony loss, starting from the numerous variables recorded in this study. No interaction effects between the 4 model factors/covariates were found.

**Fig 4:**
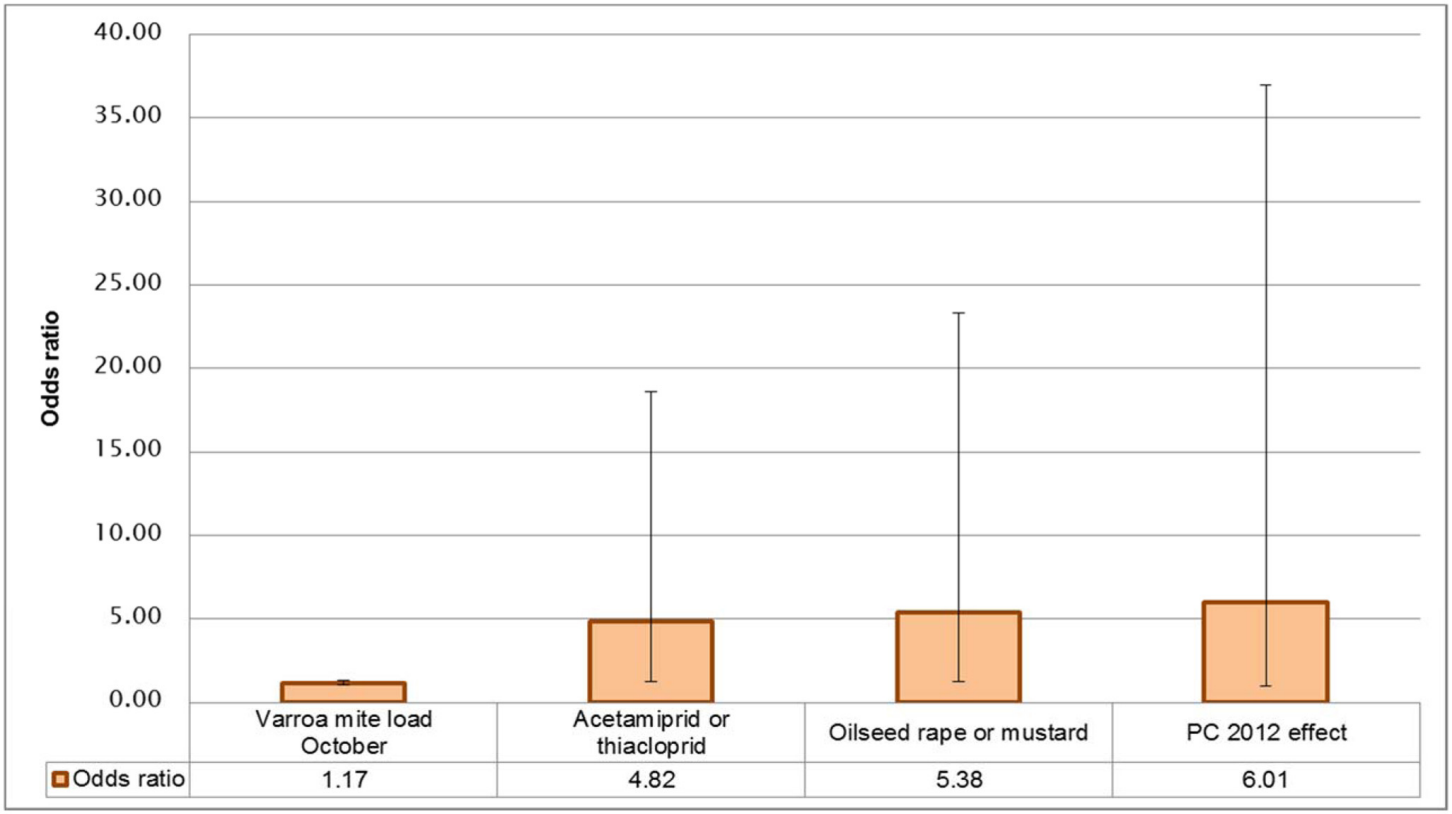
Odds Ratios for the fixed effects from the final model, with 95% confidence intervals. Fixed model terms are acetamiprid or thiacloprid present in honey, bee bread or bees in summer 2011, rape/mustard present in bee bread in summer 2011, attributed postal code random effects 2011–2012 (PC 2012 effects) and varroa mite load in October 2011. For the categorical factors, the odds ratio is the odds of loss for colonies with acetamiprid/thiacloprid present divided by the odds for colonies with these absent, and similarly for oilseed rape/mustard, i.e. the proportional increase or decrease in the odds. For the continuous terms, the odds ratio is the proportional increase in the odds of loss for an increase of 1 unit in the value of the continuous variable.

Fig 4 shows more clearly the relative sizes of the odds ratios in Table 2 for the 4 terms in the final model M1, with 95% confidence intervals. The p-values in Tables 2 and 3 indicate the importance of the individual variables in the final 4 variable model (whereas Table 1 gives a comparison of the null model, single variable models and 4 variable models). The most statistically significant term in the final model is the varroa mite rate in October, followed by contamination with the cyano-substituted neonicotinoids acetamiprid or thiacloprid (yes/no) in honey, pollen or bee matrices, then rape/mustard found (yes/no) in bee bread, with PC 2012 effect being the least significant of the predictor variables. The estimated odds ratios are about 5 times as large for the last 3 of these compared to the odds ratio of an increase of size one in the varroa mite load, however their slope parameters cannot be estimated as precisely from the available data, so the standard errors of these effects are larger than for varroa mite effect and the confidence intervals for these odds ratios are therefore much wider than for varroa mite effect (Table 2 and Fig 4). Although this appears to be a contradiction, it is a statistical consequence of the sample size available for the estimation of each effect and of the variation in the values of the covariates. Increasing the sample size in a further study may resolve this to some extent, as it should have the benefit of reducing the standard errors of all of the estimated effects in the fitted model.

## Discussion

In our model fitting we included the size of the PC 2012 area random effect as a fixed effect in a GzLM. An alternative approach would have been to fit a GLMM including PC area as a random effect estimated from the present data. Using the GzLM approach allowed us to assess the relationship between the risk of winter loss and previously unexplained area effects as estimated from a larger dataset, and also to assess the relative importance of the different terms in the model, as they are all fixed effects.

In building our final best fitting model we considered many variables and factors in single variable models, to demonstrate how we came to the most parsimonious model. In this discussion we mostly focus on the variables found to be significantly associated with the risk of colony winter loss in this best fitting model, rather than all of the variables examined during the model fitting process.

Analysis of annual Dutch questionnaire data [2–5] has shown that varroa mite treatment was the strongest factor in explaining reported honey bee colony winter losses. The outcome of the present observational study of potential risk factors at colony level confirms the important contribution of this ectoparasite to honey bee winter losses. A high varroa mite load in October, indicating failing varroa control, was the strongest factor in the best fitting model for the risk of colony winter loss. This is in line with the findings in many studies such as the German longitudinal surveillance study in [15], the pan-European study in [18], Becher et al. in [30] and a study on varroa infestation [27]. No association was found between risk of winter loss and varroa mite load in July, however in July serious varroa control measures were only started by one beekeeper. The annual Dutch Winter Survival Monitoring Surveys also show that most of the beekeepers treat the varroa mite in August and September, after harvesting the honey of the preceding month(s).

Neither the presence of the various bee viruses detected during sampling in July, nor presence of *Nosema* spp. in the colonies in July, were associated with risk of winter loss, nor was there evidence of interactions between these viruses and *Nosema* spp. and other model factors. Investigating the occurrence of these viruses in October might have given a different result, as a German study found a strong positive relation between the occurrence of the varroa mite vectored viruses DWV and ABPV (both extracted from bee heads for analysis) in October and winter loss [15]. However this was not considered in the present study, in order to limit costs.

Considering pesticides, the authors of [15] found no association between winter loss and contamination with agricultural pesticides in oilseed rape pollen (personal communication with the first author of [15]). In the present study we also found no significant evidence of such an association with pesticides, although the presence of rape/mustard in bee bread was a significant model factor which was associated with higher losses. For instance, we found no significant association with higher losses when we investigated interaction of the effects on losses of rape/mustard in bee bread and the presence of thiacloprid or acetamiprid in honey, pollen or bees. This was expected, since thiacloprid and acetamiprid were not accepted pesticides for use on oilseed rape in the Netherlands at the time. The effect of interactions of rape/mustard with other agricultural pesticides on risk of colony winter loss could not be estimated, because in this study only neonicotinoids were detected in sufficient quantities to allow for a statistical analysis. It is possible therefore that such interactions do exist and might have been detected with a larger sample of colonies than we used in this study.

Only a few colonies had neonicotinoids in only one of the matrices. We found no effect on risk of loss if neonicotinoids were only present in honey or pollen respectively compared with colonies without neonicotinoid presence. We found a strong effect on the odds of winter loss if we compared neonicotinoid presence in honey with absence of neonicotinoids in honey, while such an effect was not found for presence versus absence of neonicotinoids in pollen. Contamination of pollen with the pooled factor acetamiprid/thiacloprid compared with all the colonies in which these cyano-substituted neonicotinoids were not present was however significant, although the effect for honey was much stronger in a similar analysis. We consider this as mildly suggestive that presence of cyano-substituted neonicotinoids in honey might be the better indicator for the risk of loss rather than their presence in pollen, but more investigations are necessary to come to sound conclusions on this issue. The intake of honey per individual bee is substantially higher than the pollen intake [31] which further supports the above finding. We should however consider that in a substantial number of colonies neonicotinoids were simultaneously present in more than one matrix. These possibly confounding effects make it difficult to draw strong conclusions about the effects of these single factors, other than exploring which factors are candidates for testing in a multifactorial model.

Presence of thiacloprid or acetamiprid (yes/no) in honey bee matrices at the beginning of August was another strong factor in the best explaining model, and their presence was also associated with higher odds of winter loss. The fit of the next best explaining model indicated that presence of any neonicotinoids (yes/no) in the matrices at the start of August was almost as important as the combined factor acetamiprid/thiacloprid. Amongst thiacloprid, acetamiprid, and imidacloprid, thiacloprid was found to be the most important in explaining risk of colony loss. Thiacloprid was also the most commonly occurring neonicotinoid in this dataset.

In the first period of winter (before January 1 2012) colonies with thiacloprid present in one of the matrices had significantly higher odds of loss compared to colonies without neonicotinoids, illustrating a reduced lifespan for the contaminated colonies. To our knowledge such a result has not been found in other observational studies, which is understandable because cyano-substituted neonicotinoids are considered to have low toxicity. Iwasa et al. [32] found in laboratory studies that the cyano-substituted neonicotinoids were less toxic for bees compared to those with nitro-substitution and suggested that at least acetamiprid could be safe for bees. A possible explanation for our contrasting finding could be as suggested by Laurino et al. [33], who found in an experimental study that ‘*acetamiprid and thiacloprid*, *as also evidenced in other acute toxicity trials* [32, 34], *were apparently not dangerous to the honey bees unless they were starved*. *This result suggests that there is a repellent effect of both a*.*i*. *as also reported for imidacloprid* [35] *and a food preference test would prove such an effect*. *If so*, *and disregarding sub-lethal effects*, *some hazards can arise when colonies are severely short of stores or after prolonged seclusion’*. In fact conditions suggesting food deprivation were observed in the colonies in the present study. This may be due to unfavourable weather conditions. The Royal Netherlands Meteorological Institute [36] reported that July 2011 was the 6th wettest July since the year 1901. The temperature was 2 degrees below the long-term average, there were only 158 hours of sunshine compared to 212 hours normally, and there was a total monthly precipitation of 179 mm in 96 hours compared to the long term July average of only 81 mm of precipitation in 40 hours. So there were many more hours of rain than is usual. August 2011 showed a similar pattern, with 153 hours of sunshine, whereas 195 hours is the long term average, and 110 mm of precipitation, while only 78 mm is the long term average. These weather conditions resulted in bad foraging opportunities for honey bee colonies during the high summer period. These bad foraging conditions during the main honey flows in early summer were reflected in the low general honey and pollen reserves in the colonies observed in the first two weeks of August 2011, even though most of the beekeepers had started supplementary feeding of their colonies. As a consequence, starvation may have played a role. Also, exposure to contaminated honey and pollen may have been at a relatively high level because of reduced opportunities for colonies to detoxify the limited stored food supplies by the uptake of non-contaminated foods and so to decrease the concentration of acetamiprid or thiacloprid by diluting these ingredients [37]. We consider however such starvation and detoxification as a worst case scenario and cannot exclude the possibility that the cyano-substituted neonicotinoids had an effect irrespective of the nutritional status of the bees as was found by Retschnig et al. [38]. They found higher mortality of individual bees in colonies fed with thiacloprid (and the pyrethroid tau-fluvalinate) compared with the control group at one of two locations. It is also possible that factors (such as other pesticides or heavy metals) may be observed in future research which may also explain or better explain the effects on loss which are now attributed to thiacloprid.

Some colonies were in a position to compensate for these bad high summer weather circumstances. September, October and November were warmer and drier than normal. The analysis of the Dutch National Monitoring Survey 2012 [4] showed that colonies with access to *Calluna vulgaris* honey flow in August 2011 and September 2011 had lower odds of winter loss compared with colonies without this access (p<0.0001). Relatively warm weather continued in October and November, which provided these colonies with opportunities to produce a winter bee population in late summer. An indication of such an effect of lower odds of loss (p = 0.0954) was found in the present study sample for the limited number of colonies (12 colonies wintered, 1 lost) with *Calluna vulgaris* pollen present in bee bread. The presence of *Calluna vulgaris* in bee bread was also significantly (p = 0.0087) associated with PC areas with lower, more negative values of the PC random effect. *Calluna* is only available in specific areas of the Netherlands, and blossoms in late summer. There is therefore an indication towards a positive effect of *Calluna* presence on colony losses, and *Calluna* presence can help to explain a lower risk of loss in some areas.

In the Dutch National Survey 2011 van der Zee and Pisa [3] found an effect of higher odds of loss for apiaries of beekeepers who stated that their colonies were in a position to forage on maize. Maize was present within a distance of 3 km of the apiaries of 94% of 1571 participating Dutch beekeepers in the National Survey 2014 (unpublished data, van der Zee). Wille and Wille [39] and Wille et al. [40] found that maize was one of the main providers of pollen for honeybees in some Swiss regions. Possible effects of seed-coated maize were not clear, as was mentioned in the 2012 Survey report [4]. Also, no such effect was found in the National Survey 2011 [3]. In the present study we found that the presence of maize in bee bread was not associated with the risk of colony loss in the observed colonies, but was significantly (p = 0.0131) associated with PC areas with lower, more negative values of the PC random effect. Use of maize seed coated with neonicotinoids was low (about 8%) in 2011 as we were informed by the Land en Tuinbouw Organisatie Noord (The Dutch farmer union LTO North) based on information derived from Agrodis, the organisation which represents the producers of agricultural pesticides in the Netherlands. In 2012 9–10% of the total area used for maize production was coated with (mainly) thiamethoxam and clothianidin and a limited amount of imidacloprid, and in 2013 13% of the maize-producing area was coated. It was mostly used in the Northern parts of the Netherlands where maize cultivation is rotated with grassland and neonicotinoids are used as treatment against *Coleoptera* larvae (mainly present in grassland). Such information is however not precise enough to investigate associations between presence of coated maize in specific postal code areas and higher risk of winter losses in the present study population.

Whilst the presence of rape/mustard in bee bread was significantly associated with higher risk of loss, it was not associated with the PC random effect. This may be because this forage source, rape at least, is grown in particular areas, but many beekeepers traditionally migrate their colonies to these areas to profit from the large rape nectar flow.

The PC area effect, calculated from the Dutch National Monitoring Survey 2012 and attributed to the colonies in the present study as a fixed model covariate, was the least statistically significant term in the model which best explained the loss data. This may be expected because the postal code areas are not ecologically defined, but are administrative entities. However, variation in risk of loss between postal code areas may still be expected, because the type of soil mainly determines land use and stretches over larger surfaces than postal code areas.

Spatial variation in the study sample may be partly due to differences in mite load between PC areas, since higher varroa mite infestation rates in October were correlated (p = 0.0069) with higher, more positive, values of the attributed PC effect. Similarly, colonies with acetamiprid or thiacloprid were associated with PC areas with higher more positive values of the attributed PC effect (p = 0.0265). This was expected since crops are mostly present in these higher risk postal codes areas, while land use in the lower risk postal code areas is mostly grassland and uncultivated areas (see Fig 2.2 showing dominant land use in [41]). We conclude that the regional variation in attributed postal code effects may to some extent be explained by variation in mite loads and acetamiprid and thiacloprid presence in colonies, and that higher mite loads in October and presence of these pesticides is associated with a greater risk of winter loss.

Whilst the beekeepers were asked not to migrate their bees during the study period, some of them may have migrated their bees earlier in the season, to other areas. Any such migration may be expected to weaken the area effect. Migrated colonies may have been exposed to influences which affected colony health, positively or negatively. Therefore the area effect in our modelling would be influenced for part of the foraging season by effects from other areas than the area in which the colony is normally kept. A study over more years could help to clarify this issue. We found that the area effect was significant in all the various multivariable models which we fitted. Omitting the PC area effect from the multivariable model containing thiacloprid as a single pesticide factor led to thiacloprid presence becoming more significant. This further suggests a link between thiacloprid presence and area. That is, thiacloprid presence is partially explained by area. This was also true for the pooled factor acetamiprid/thiacloprid but not for acetamiprid alone nor for imidacloprid nor the “any neonicotinoids” factor.

It could be argued that using the PC 2012 effects as a measure of variation in unexplained losses between areas as a predictor of losses in winter 2011–2012 in the present study is not appropriate since these PC 2012 effects also relate to losses over winter 2011–2012. They were derived from a different larger-scale dataset, although the beekeepers in this study were also included in the larger study. However, the PC 2012 effects from the National Monitoring Survey 2012 provide a reliable measure of the risk of loss in each PC area which could not be explained by variables recorded in the beekeeper questionnaires. The analysis of the Dutch Monitoring Surveys [2–5] confirms that these effects are consistent from year to year in identifying areas where important causes of loss remain to be explained. The PC 2012 effects were the most up-to-date estimates of these effects available that could be used in the modelling here. The PC random effects from the Dutch National Monitoring Survey 2011, which we used to divide apiaries into those in higher and lower risk areas for sample selection, could have been used in the model fitting instead of those from the 2012 Monitoring Survey. Not surprisingly, these PC 2011 and PC 2012 effects are significantly positively correlated (for the 75 colonies included in the final modelling the Spearman correlation is 0.631, with a near zero p-value). Either could therefore be used a covariate in the final model. We did in fact try using the PC 2011 effects in the model fitting instead. It made very little difference to the final model fit and the conclusions. The relative importance of the variables in the model was the same as when using the PC 2012 effects. However the PC 2011 effects were calculated from a model which only included an intercept, a beekeeper random effect and a postal code random effect, rather than a model including explanatory variables as well. Therefore the PC 2011 effects are an overall measure of variation in risk of winter loss between PC areas, relating to losses in winter 2010–2011, rather than variation in unexplained risk of loss. We feel that use of the PC 2012 effects as a measure of unexplained variation in risk of loss between PC areas is more appropriate. The similarity in the relation between location (postal code effects) and risk of loss in 2011 and 2012 indicates that spatial factors may well play a role. These factors have not been identified in the Dutch Winter Survival Monitoring Surveys, but might be related to land use, as the present study suggests.

Adding information on land use to the modelling might enable replacing postal code areas by a better explaining spatial effect, which will be crucial for further exploration and understanding of local effects. Using database information on land use and use of pesticide on agricultural crops may also be helpful in the modelling of data from the annual National Monitoring Survey (data collection from beekeepers via questionnaires). This approach might also allow for a better understanding of increases and decreases of other pollinators also potentially affected by land use and agricultural pesticides.

In a recent publication [42] healthy/not healthy colonies, rather than not lost/lost colonies, were compared in a small-scale observational study of colonies in Belgium, over the same time frame as in our present study, namely summer 2011, with an evaluation of colony health in spring 2012. The authors found a significant association between increase in risk of ‘colony disorder’ and increasing extent of land used for crops around the apiary, whereas we found odds of higher winter loss in postcode areas with more positive postcode random effects. The Belgian finding in relation to land use is interesting and reinforces the need to incorporate land use information in further model building. We disagree with their remark that the bee disorders happened despite normal climatic conditions. They only consider weather in the period October-December 2011, which was dry and sunny as in the Netherlands. The weather in summer [43] was also comparable with that in the Netherlands and was very unfavourable for foraging. This may have had an impact not only on losses discussed in the present study, but also on the outcome of this Belgian study.

In another, much larger, Belgian study by Ravoet et al. [44] samples from an earlier study were re-analysed for the prevalence of the newly discovered trypanosomid parasite *C*. *mellificae* together with 16 known honeybee pathogens. The samples, which originated from 363 colonies, were collected in July 2011 (comparable with the time of sampling in our study). In their study 46.5% of colonies were lost over winter 2011–2012. Ravoet et al. also used a generalized linear modelling approach to relate the presence of these pathogens in summer to winter loss. In their final model using data from 229 colonies, presence of *C*. *mellificae* and *N*. *ceranae* were both significant factors explaining winter loss. *V*. *destructor* had a p-value of 0.07 in the model, reported as significant also. They also found an interaction effect of *C*. *mellificae* with *N*. *ceranae*, significantly contributing in this model to explain winter mortality. *C*. *mellificae* may have played a role in our study too, but was not one of the pathogens which we considered. *Nosema ceranae* was not an explaining factor in our study nor in most Northern European countries [38, 44]. These sometimes contrasting outcomes demonstrate that honey bee losses can only be understood by considering a range of pathogens and pesticides which may only contribute to losses under specific conditions.

Our study was a relatively small scale national exercise undertaken as a first exploration to investigate the presence of several agricultural pesticides in Dutch honey bee matrices and their effects on winter loss, involving colony losses and colony samples collected in the summer of a single year. Consequently, the data and conclusions here are limited to the Netherlands. A longitudinal study over more years, with more colonies, and ideally more pesticides, is necessary to further clarify the findings in this study.

## Conclusions

This study illustrates the vulnerability of honey bees to winter loss when several factors affecting health reduce the chance of producing a strong winter population in high summer. In the model fitting of the data, the most statistically significant term was the varroa mite rate in October, followed by presence/absence of acetamiprid or thiacloprid in honey, pollen or bee matrices, then presence/absence of rape/mustard in bee bread, with the postcode area effect being the least significant term in the model. The estimated odds ratios were about 5 times as large for the neonicotinoids, rape/mustard and postcode area effect than the odds ratio for increasing varroa mite load by one unit, but in the fitted model the standard errors of the slope parameters for these first 3 effects were much larger than for varroa mite effect. This means that although the sizes of the effects of acetamiprid/thiacloprid and rape/mustard in particular appear to be much larger than that of varroa mite load, they are not as statistically significant and do not explain as much of the variation in the loss rates. Increasing the sample size in a future study would have the benefit of reducing the standard errors of all of the estimated effects in the fitted model, which should give a clearer view of the relative importance of the effects on the risk of winter colony loss and may also reveal interactions between model factors not found in the present study.

Bad weather may have played an important role in the observed losses, because reduced foraging opportunities in July and August may have impacted adversely on the production of a healthy winter population, not only by deprivation of the necessary food supply, but also because starvation may have increased the toxic effects of thiacloprid and acetamiprid. These effects may be playing a role in areas with reduced food sources in summer under less extreme weather conditions also.

## Supporting Information

S1 Dataset Excel data spreadsheet. Data for all the colonies for re-creation of the significant results.
(XLSX)

S2 Dataset Excel data spreadsheet. Data for the 75 colonies used in the final model fitting with the relevant variables.
(XLSX)

S1 File Text file which explains the definition of the variables in the S1 and S2 Datasets. (TXT)

S1 Table Reference material for pesticides. (DOCX)

S2 Table List of analytes with MS/MS parameters. (DOCX)

S3 Table Validation for Honey. (DOCX)

S4 Table Validation for Bees. (DOCX)

S5 Table Validation for Stored Pollen. (DOCX)

S6 Table Number of analysed samples per matrix for each component. (DOCX)
