# Supplementary material for: An Observational Study of Honey Bee Colony Winter Losses and Their Association with Varroa destructor, Neonicotinoids and Other Risk Factors
Source: PLoS One. 2015 Jul 8;10(7):e0131611. doi: 10.1371/journal.pone.0131611 (PMC4496033; doi:10.1371/journal.pone.0131611)
Supplement: S1 Table — (DOCX) [file pone.0131611.s004.docx]

Table S1. Reference material for pesticides

| Pesticide |  |  | Stock solution | | |
| --- | --- | --- | --- | --- | --- |
|  | Origin | Purity | Concentration | Solvent^(1)^ | Concentration mix |
|  |  | % | ug/ml |  | (µg/ml) |
| 6-Chloronicotinic acid | S | 99.0 | 2000 | MeOH | 20 |
| Acetamiprid | S | 99.9 | 2000 | MeOH | 1.0 |
| Clothianidin | S | 99.9 | 2000 | MeOH | 4.0 |
| Coumaphos | E | 96.0 | 2000 | ACN | 4.0 |
| DMA | S | 99.0 | 2000 | MeOH | 50 |
| DMF | S | 97.0 | 2000 | MeOH | 10 |
| DMPF | S | 99.7 | 500 | MeOH | 10 |
| Fipronil | E | 98.0 | 2000 | MeOH | 1.0 |
| Fipronil-sulfone | E | 99.5 | 2000 | ACN | 1.0 |
| Fipronil-carboxamide | E | 96.5 | 100 | ACN | 1.0 |
| Fipronil-desulfinyl | E | 96.5 | 100 | ACN | 1.0 |
| Fipronil-sulfide | E | 99.5 | 1000 | MeOH | 1.0 |
| Fluvalinate-tau | E | 91.0 | 2000 | ACN | 20 |
| Imidacloprid | E | 99.0 | 2000 | MeOH | 1.0 |
| Imidacloprid olefin | B | 97.9 | 500 | MeOH | 10 |
| Imidacloprid urea | B | 99.4 | 500 | MeOH | 1.0 |
| Imidacloprid, 5-hydroxy | B | 96.7 | 500 | MeOH | 10 |
| Imidacloprid, desnitro | B | 97.9 | 500 | MeOH | 1.0 |
| Imidacloprid, desnitro olefin | B | 99.9 | 500 | MeOH | 1.0 |
| Piperonyl butoxide | E | 92.5 | 2000 | ACN | 1.0 |
| Propiconazole | E | 97.0 | 2000 | MeOH | 10 |
| Thiacloprid | E | 99.5 | 1000 | MeOH | 2.0 |
| Thiamethoxam | E | 99.5 | 2000 | MeOH | 4.0 |
| Triflumizole | S | 99.8 | 2000 | MeOH | 2.0 |

^(1)^ ACN = acetonitrile; MeOH = methanol
