# Supplementary material for: An Observational Study of Honey Bee Colony Winter Losses and Their Association with Varroa destructor, Neonicotinoids and Other Risk Factors
Source: PLoS One. 2015 Jul 8;10(7):e0131611. doi: 10.1371/journal.pone.0131611 (PMC4496033; doi:10.1371/journal.pone.0131611)
Supplement: S2 Table — (DOCX) [file pone.0131611.s005.docx]

Table S2. List of analytes with MS/MS parameters

| Pesticide | m/z Precursor ion | DP (V) | m/z Product ions | CE (V) | CXP (V) |
| --- | --- | --- | --- | --- | --- |
| 6-Chloronicotinic acid | 155.9 | -50 | 111.9 | -14 | -13 |
| Acetamiprid | 223 | 51 | 126.0/73.0 | 29/79 | 10/12 |
| Clothianidin | 249.9 | 46 | 169.1/132.0 | 17/23 | 12/10 |
| Coumaphos | 363 | 85 | 227.0/307.0 | 35/23 | 15/15 |
| DMA | 122 | 91 | 107.1/77.1 | 23/37 | 8/10 |
| DMF | 150 | 41 | 107.1/77.0 | 27/53 | 8/12 |
| DMPF | 163 | 76 | 122.1/107.2 | 23/33 | 8/8 |
| Fipronil | 434.9 | -50 | 329.7/249.9 | -18/-36 | -21/-17 |
| Fipronil-carboxamide | 452.9 | -80 | 348.0/303.8 | -22/-34 | -23/-21 |
| Fipronil-desulfinyl | 387 | -50 | 350.8/281.8 | -20/-44 | -25/-17 |
| Fipronil-sulfide | 418.9 | -40 | 261.9/382.8 | -40/-16 | -17/-11 |
| Fipronil-sulfone | 450.9 | -30 | 281.9/414.7 | -40/-22 | -19/-29 |
| Fluvalinate-tau | 503 | 30 | 181.0/208.1 | 35/17 | 12/14 |
| Imidacloprid | 256.1 | 41 | 209.0/175.1 | 21/25 | 14/12 |
| Imidacloprid olefin | 253.9 | 56 | 171.2/204.9 | 25/21 | 12/14 |
| Imidacloprid urea | 212.2 | 41 | 128.0/99.1 | 25/25 | 10/8 |
| Imidacloprid, 5-hydroxy | 271.9 | 51 | 146.1/225.1 | 39/23 | 10/16 |
| Imidacloprid, desnitro | 210.9 | 76 | 90.1/72.9 | 47/77 | 8/6 |
| Imidacloprid, desnitro olefin | 208.9 | 66 | 126.0/73.0 | 29/67 | 10/8 |
| Piperonyl butoxide | 356.2 | 31 | 177.1/119.1 | 19/47 | 25/25 |
| Propiconazole | 342 | 106 | 159.0/69.1 | 37/23 | 10/6 |
| Thiacloprid | 253 | 90 | 126.0/186.0 | 29/19 | 15/15 |
| Thiamethoxam | 292 | 41 | 210.9/180.9 | 17/31 | 14/12 |
| Triflumizole | 346 | 26 | 278.0/73.1 | 15/21 | 18/10 |
