# Supplementary material for: An Observational Study of Honey Bee Colony Winter Losses and Their Association with Varroa destructor, Neonicotinoids and Other Risk Factors
Source: PLoS One. 2015 Jul 8;10(7):e0131611. doi: 10.1371/journal.pone.0131611 (PMC4496033; doi:10.1371/journal.pone.0131611)
Supplement: S3 Table — (DOCX) [file pone.0131611.s006.docx]

Table S3. Validation for Honey

| **Pesticide** |  | **Recovery** | | | | | | | | |
| --- | --- | --- | --- | --- | --- | --- | --- | --- | --- | --- |
|  | SL | 1x SL | | | 2x SL | | | 10x SL | | |
|  | (µg/kg) | N | Avg.(%) | RSD(%) | n | Avg.(%) | RSD(%) | n | Avg.(%) | RSD(%) |
| 6-Chloronicotinic acid | 10 | 10 | 26 | 15 | 8 | 23 | 21 | 10 | 22 | 19 |
| Acetamiprid | 0.50 ^(1)^ | 10 | 94 | 4.5 | 8 | 94 | 4.6 | 10 | 90 | 5.7 |
| Clothianidin | 2.0 | 10 | 92 | 9.3 | 8 | 93 | 5.7 | 10 | 92 | 5.3 |
| Coumaphos | 2.0 ^(2)^ | 8 | 92 | 6.7 | 6 | 90 | 6.8 | 8 | 88 | 7.6 |
| DMA | 25 | 10 | 82 | 12 | 8 | 85 | 7.6 | 10 | 88 | 14 |
| DMF | 5.0 | 10 | 88 | 5.0 | 8 | 89 | 5.5 | 10 | 87 | 3.9 |
| DMPF | 5.0 ^(3)^ | 10 | 89 | 4.0 | 8 | 89 | 5.9 | 10 | 65 | 20 |
| Fipronil | 0.50 | 10 | 108 | 15 | 8 | 99 | 12 | 10 | 103 | 7.2 |
| Fipronil-carboxamide | 0.50 | 10 | 94 | 8.3 | 8 | 92 | 7.4 | 10 | 96 | 3.0 |
| Fipronil-desulfinyl | 0.50 | 10 | 104 | 12 | 8 | 95 | 6.9 | 10 | 98 | 4.3 |
| Fipronil-sulfide | 0.50 | 10 | 106 | 15 | 8 | 95 | 9.2 | 10 | 101 | 5.0 |
| Fipronil-sulfone | 0.50 | 10 | 99 | 12 | 8 | 92 | 7.9 | 10 | 96 | 3.5 |
| Fluvalinate-tau | 10 ^(4)^ | 10 | 78 | 6.8 | 8 | 79 | 5.9 | 10 | 82 | 10 |
| Imidacloprid | 0.50 ^(5)^ | 10 | 90 | 14 | 8 | 91 | 7.1 | 10 | 91 | 3.2 |
| Imidacloprid olefin | 5.0 | 10 | 96 | 10 | 8 | 94 | 13 | 10 | 94 | 5.4 |
| Imidacloprid urea | 0.50 | 10 | 93 | 5.7 | 8 | 92 | 3.7 | 10 | 91 | 3.9 |
| Imidacloprid 5-hydroxy | 5.0 | 10 | 94 | 2.8 | 8 | 92 | 6.1 | 10 | 91 | 5.0 |
| Imidacloprid, desnitro | 0.50 | 10 | 70 | 4.2 | 8 | 67 | 4.6 | 10 | 95 | 6.8 |
| Imidacloprid, desnitro olefin | 0.50 | 10 | 74 | 5.2 | 8 | 73 | 5.4 | 10 | 91 | 3.8 |
| Piperonyl-butoxide | 0.50 | 8 | 85 | 3.3 | 6 | 82 | 3.3 | 8 | 84 | 2.9 |
| Propiconazole | 5.0 ^(6)^ | 10 | 96 | 3.5 | 8 | 93 | 5.0 | 10 | 93 | 4.4 |
| Thiacloprid | 1.0 ^(7)^ | 10 | 93 | 2.5 | 8 | 93 | 6.1 | 10 | 91 | 5.4 |
| Thiamethoxam | 2.0 | 10 | 95 | 5.4 | 8 | 94 | 5.0 | 10 | 92 | 4.8 |
| Triflumizole | 1.0 | 10 | 93 | 4.1 | 8 | 92 | 5.9 | 10 | 91 | 5.1 |

^(1)^ LOD = 0.20 µg/kg, ^(2)^ LOD = 1.5 µg/kg, ^(3)^ LOD = 2.5 µg/kg, ^(4)^ LOD = 4.0 µg/kg, ^(5)^ LOD = 0.30 µg/kg, ^(6)^ LOD = 1.0 µg/kg, ^(7)^ LOD = 0.25 µg/kg
