# Supplementary material for: An Observational Study of Honey Bee Colony Winter Losses and Their Association with Varroa destructor, Neonicotinoids and Other Risk Factors
Source: PLoS One. 2015 Jul 8;10(7):e0131611. doi: 10.1371/journal.pone.0131611 (PMC4496033; doi:10.1371/journal.pone.0131611)
Supplement: S4 Table — (DOCX) [file pone.0131611.s007.docx]

Table S4. Validation for Bees

| Pesticide |  | Recovery | | | | | | | | |
| --- | --- | --- | --- | --- | --- | --- | --- | --- | --- | --- |
|  | SL | 1x SL | | | 2x SL | | | 10x SL | | |
|  | (µg/kg) | n | Avg. (%) | RSD (%) | n | Avg. (%) | RSD (%) | n | Avg. (%) | RSD (%) |
| 6-Chloronicotinic acid | 10 | 4 | 29 | 9.5 | 4 | 30 | 20 | 4 | 27 | 15 |
| Acetamiprid | 0.50 ^(1)^ | 8 | 85 | 9.1 | 8 | 83 | 9.5 | 6 | 79 | 9.7 |
| Clothianidin | 2.0 | 8 | 88 | 26 | 8 | 93 | 18 | 6 | 80 | 23 |
| Coumaphos | 2.0 ^(2)^ | 8 | 81 | 3.3 | 8 | 84 | 6.0 | 6 | 82 | 11 |
| DMA | 25 | 8 | 61 | 17 | 8 | 73 | 8.6 | 6 | 76 | 16 |
| DMF | 5.0 | 8 | 86 | 8.9 | 8 | 83 | 6.6 | 6 | 87 | 9.2 |
| DMPF | 5.0 | 8 | 84 | 3.8 | 8 | 87 | 6.1 | 6 | 49 | 7.5 |
| Fipronil | 0.50 | 4 | 81 | 15 | 4 | 85 | 6.9 | 4 | 82 | 3.7 |
| Fipronil-carboxamide | 0.50 | 8 | 84 | 7.6 | 8 | 89 | 3.4 | 6 | 87 | 12 |
| Fipronil-desulfinyl | 0.50 | 4 | 78 | 5.6 | 4 | 79 | 5.0 | 4 | 86 | 9.3 |
| Fipronil-sulfide | 0.50 | 4 | 86 | 8.3 | 4 | 89 | 5.7 | 4 | 84 | 4.7 |
| Fipronil-sulfone | 0.50 | 4 | 88 | 9.6 | 4 | 90 | 4.6 | 4 | 86 | 3.7 |
| Fluvalinate-tau | 10 | 8 | 74 | 25 | 8 | 76 | 22 | 6 | 84 | 11 |
| Imidacloprid | 0.50 ^(3)^ | 8 | 102 | 11 | 8 | 96 | 12 | 6 | 98 | 16 |
| Imidacloprid olefin | 5.0 | 8 | 102 | 16 | 8 | 107 | 20 | 6 | 105 | 23 |
| Imidacloprid urea | 0.50 | 8 | 86 | 6.6 | 8 | 87 | 6.6 | 6 | 87 | 13 |
| Imidacloprid, 5-hydroxy | 5.0 | 8 | 112 | 21 | 8 | 118 | 34 | 6 | 113 | 30 |
| Imidacloprid, desnitro | 0.50 ^(4)^ | 8 | 67 | 3.8 | 8 | 67 | 8.9 | 6 | 86 | 5.0 |
| Imidacloprid, desnitro olefin | 0.50 | 8 | 68 | 2.2 | 8 | 70 | 10 | 6 | 80 | 4.9 |
| Piperonyl-butoxide | 0.50 | 8 | 79 | 8.7 | 8 | 79 | 17 | 6 | 86 | 6.1 |
| Propiconazole | 5.0 ^(5)^ | 8 | 82 | 4.9 | 8 | 86 | 5.1 | 6 | 84 | 13 |
| Thiacloprid | 1.0 ^(6)^ | 8 | 90 | 18 | 8 | 92 | 14 | 6 | 83 | 12 |
| Thiamethoxam | 2.0 | 8 | 94 | 15 | 8 | 95 | 17 | 6 | 82 | 18 |
| Triflumizole | 1.0 | 8 | 83 | 4.3 | 8 | 85 | 5.0 | 6 | 81 | 8.9 |

^(1)^ LOD = 0.20 µg/kg, ^(2)^ LOD = 0.40 µg/kg, ^(3)^ LOD = 0.30 µg/kg, ^(4)^ LOD = 0.20 µg/kg, ^(5)^ LOD = 2.0 µg/kg, ^(6)^ LOD = 0.20 µg/kg
