# Supplementary material for: An Observational Study of Honey Bee Colony Winter Losses and Their Association with Varroa destructor, Neonicotinoids and Other Risk Factors
Source: PLoS One. 2015 Jul 8;10(7):e0131611. doi: 10.1371/journal.pone.0131611 (PMC4496033; doi:10.1371/journal.pone.0131611)
Supplement: S5 Table — (DOCX) [file pone.0131611.s008.docx]

Table S5. Validation for Stored Pollen

| Pesticide |  | Recovery | | | | | | | | |
| --- | --- | --- | --- | --- | --- | --- | --- | --- | --- | --- |
|  | SL | 1x SL | | | 2x SL | | | 10x SL | | |
|  | (µg/kg) | n | Avg. (%) | RSD (%) | n | Avg. (%) | RSD (%) | n | Avg. (%) | RSD (%) |
| 6-Chloronicotinic acid | 10 | 6 | 21 | 8.5 | 6 | 21 | 14 | 6 | 21 | 7.1 |
| Acetamiprid | 0.50^(1)^ | 6 | 95 | 16 | 6 | 82 | 5.8 | 6 | 82 | 4.7 |
| Clothianidin | 2.0 | 6 | 85 | 13 | 6 | 85 | 10 | 6 | 80 | 11 |
| Coumaphos | 2.0^(2)^ | 6 | 85 | 3.0 | 6 | 80 | 6.8 | 6 | 84 | 2.4 |
| DMA | 25 | 6 | 61 | 9.8 | 6 | 62 | 6.8 | 6 | 68 | 4.3 |
| DMF | 5.0^(3)^ | 6 | 105 | 21 | 6 | 86 | 12 | 6 | 90 | 7.6 |
| DMPF | 5.0 | 6 | 74 | 4.2 | 6 | 74 | 7.8 | 6 | 45 | 10 |
| Fipronil | 0.50 | 6 | 78 | 9.7 | 6 | 80 | 18 | 6 | 81 | 9.0 |
| Fipronil-carboxamide | 0.50 | 6 | 90 | 7.2 | 6 | 83 | 15 | 6 | 91 | 9.1 |
| Fipronil-desulfinyl | 0.50 | 6 | 70 | 20 | 6 | 76 | 5.7 | 6 | 84 | 4.6 |
| Fipronil-sulfide | 0.50 | 6 | 85 | 4.5 | 6 | 81 | 9.4 | 6 | 86 | 3.9 |
| Fipronil-sulfone | 0.50 | 6 | 77 | 10 | 6 | 76 | 8.7 | 6 | 87 | 4.5 |
| Fluvalinate-tau | 10 | 6 | 73 | 21 | 6 | 61 | 25 | 6 | 70 | 10 |
| Imidacloprid | 0.50^(4)^ | 2 | 128 | 28 | 6 | 129 | 30 | 6 | 91 | 17 |
| Imidacloprid olefin | 5.0 | 6 | 85 | 12 | 6 | 80 | 9.0 | 6 | 81 | 4.3 |
| Imidacloprid urea | 0.50^(5)^ | 6 | 61 | 40 | 6 | 87 | 9.4 | 6 | 82 | 16 |
| Imidacloprid, 5-hydroxy | 5.0 | 6 | 79 | 15 | 6 | 78 | 13 | 6 | 78 | 6.9 |
| Imidacloprid, desnitro | 0.50^(6)^ | 6 | 50 | 10 | 6 | 51 | 6.0 | 6 | 64 | 2.6 |
| Imidacloprid, desnitro olefin | 0.50 | 6 | 44 | 19 | 6 | 40 | 6.6 | 6 | 53 | 1.3 |
| Piperonyl-butoxide | 0.50 | 6 | 100 | 21 | 6 | 78 | 14 | 6 | 77 | 5.8 |
| Propiconazole | 5.0^(7)^ | 6 | 81 | 5.5 | 6 | 78 | 8.4 | 6 | 85 | 3.5 |
| Thiacloprid | 1.0^(8)^ | 6 | 55 | 52 | 6 | 69 | 29 | 6 | 73 | 3.0 |
| Thiamethoxam | 2.0 | 6 | 81 | 6.8 | 6 | 86 | 9.1 | 6 | 86 | 3.7 |
| Triflumizole | 1.0 | 6 | 82 | 3.6 | 6 | 78 | 5.8 | 6 | 83 | 2.9 |

^(1)^ LOD = 0.25 µg/kg, ^(2)^ LOD = 1.0 µg/kg, ^(3)^ LOD = 4.0 µg/kg, ^(4)^ LOD = 0.75 µg/kg; LOQ = 1.0 µg/kg, ^(5)^ LOQ = 1.0 µg/kg, ^(6)^ LOD = 0.20 µg/kg, ^(7)^ LOD = 2.5 µg/kg, ^(8)^ LOD = 0.80 µg/kg; LOQ = 2.0 µg/kg
