# Supplementary material for: An Observational Study of Honey Bee Colony Winter Losses and Their Association with Varroa destructor, Neonicotinoids and Other Risk Factors
Source: PLoS One. 2015 Jul 8;10(7):e0131611. doi: 10.1371/journal.pone.0131611 (PMC4496033; doi:10.1371/journal.pone.0131611)
Supplement: S6 Table — (DOCX) [file pone.0131611.s009.docx]

Table S6. Number of analysed samples per matrix for each component

| Component | Honeybees | | | Stored pollen | | | Honey | | |
| --- | --- | --- | --- | --- | --- | --- | --- | --- | --- |
|  | Samp^(1)^ | Pos^(2)^ | Range^(3)^ | Samp^(1)^ | Pos^(2)^ | Range^(3)^ | Samp^(1)^ | Pos^(2)^ | Range^(3)^ |
| 6-Chloronicotinic acid | 42 | 0/0 | na | 42 | 0/0 | na | 42 | 0/0 | na |
| Acetamiprid | 84 | 1/1 | 0.52 | 79 | 2/3 | 0.65-5.2 | 80 | 1/6 | 0.8-3.7 |
| Clothianidin | 43 | 0/0 | na | 42 | 0/0 | na | 42 | 0/0 | na |
| Coumaphos | 84 | 2/2 | 5.0-12 | 79 | 9/5 | 4.1-26 | 80 | 0/2 | 2.3-2.7 |
| DMA ^(4)^ | 43 | 0/0 | na | 42 | 0/0 | na | 42 | 0/0 | na |
| DMF ^(5)^ | 84 | 0/0 | na | 79 | 0/1 | 6.2 | 80 | 0/2 | 17.8-22 |
| DMPF ^(6)^ | 84 | 0/0 | na | 79 | 0/2 | 7.6-9.8 | 80 | 1/1 | 6.4 |
| Fipronil | 84 | 0/0 | na | 79 | 0/2 | 2.1-2.9 | 80 | 0/0 | na |
| Fipronil carboxamide | 43 | 0/0 | na | 42 | 0/0 | na | 42 | 0/0 | na |
| Fipronil desulfinyl | 43 | 0/0 | na | 42 | 0/1 | 0.9 | 42 | 0/0 | na |
| Fipronil sulfide | 43 | 0/0 | na | 42 | 0/0 | na | 42 | 0/0 | na |
| Fipronil sulfone | 43 | 0/0 | na | 42 | 0/0 | na | 80 | 0/0 | na |
| Fluvalinate-tau | 84 | 0/0 | na | 79 | 0/2 | 13-23 | 80 | 0/0 | na |
| Imidacloprid | 84 | 2/0 | na | 79 | 3/1 | 1.1 | 80 | 2/2 | 1.0 |
| Imidacloprid olefin | 43 | 0/0 | na | 42 | 0/0 | na | 42 | 0/0 | na |
| Imidacloprid urea | 43 | 0/0 | na | 42 | 0/0 | na | 42 | 0/0 | na |
| Imidacloprid, 5-hydroxy | 43 | 0/0 | na | 42 | 0/0 | na | 42 | 0/0 | na |
| Imidacloprid, desnitro | 84 | 0/1 | 0.65 | 79 | 5/0 | na | 80 | 0/0 | na |
| Imidacloprid, desnitro olefin | 43 | 0/0 | na | 42 | 0/0 | na | 42 | 0/0 | na |
| Piperonyl butoxide | 84 | 0/0 | na | 79 | 0/6 | 0.5-2.3 | 80 | 0/0 | na |
| Propiconazole | 84 | 2/0 | na | 78 | 0/2 | 8.3-371 | 80 | 0/0 | na |
| Thiacloprid | 84 | 5/5 | 1.1-3.8 | 79 | 0/19 | 1.0-66 | 80 | 8/13 | 1.4-15 |
| Thiamethoxam | 43 | 0/0 | na | 42 | 0/0 | na | 42 | 0/0 | na |
| Triflumizole | 43 | 0/0 | na | 42 | 0/0 | na | 42 | 0/0 | na |

^(1)^ Number of analysed samples, ^( 2)^ Number of positive samples: >LOD< LOQ (trace amount) / >LOQ, ^(3)^ range of positive samples >LOQ in µg/kg, ^(4)^ amitraz metabolite: dimethylaniline, ^(5)^ amitraz metabolite: dimethylphenylformamide, ^(6)^ amitraz metabolite: dimethylphenyl-N-methylformamide, na: not available
